# Construction of a Novel Degradation Model of *Bacillus thuringiensis* Protein in Soil and Its Application in Estimation of the Degradation Dynamics of Bt-Cry1Ah Protein

**DOI:** 10.3389/fpls.2022.875020

**Published:** 2022-04-13

**Authors:** Zhilei Jiang, Lei Zhou, Baifeng Wang, Junqi Yin, Fengci Wu, Daming Wang, Liang Li, Xinyuan Song

**Affiliations:** ^1^Jilin Provincial Key Laboratory of Agricultural Biotechnology, Agro-Biotechnology Research Institute, Jilin Academy of Agricultural Sciences, Changchun, China; ^2^Institute of Quality Standard and Testing Technology for Agro-products, Chinese Academy of Agricultural Sciences, Beijing, China

**Keywords:** genetically modified crop, maize, Bt-Cry1Ah protein, environmental risk, degradation model

## Abstract

*Bacillus thuringiensis* (Bt) protein expressed by genetically modified (GM) crops is released into the soil ecosystem, where it accumulates for a long time; therefore, degradation of Bt protein has gained increased attention for environmental risk assessments. A first-order kinetic model (*Y* = ae^−b*X^) is usually used to evaluate the degradation of Bt proteins, including Bt-Cry1Ab and Bt-Cry1Ac; this has some limitations regarding the precise fitting and explanation of the influence of various factors on Bt protein degradation in the later stage. Therefore, to amend these limitations, we report a new degradation model *Y* = Y_0_ + ae^−b*X^. The effects of soil temperature, water content, soil types, and soil sterilization on the degradation of Bt-Cry1Ah protein in soil were estimated in a 96d long laboratory study using a GM maize leaf–soil mixture. The results showed that the Bt-Cry1Ah protein degraded rapidly in the early stage and then slowly in the middle and late stages. Temperature was identified as the key factor affecting the degradation of Cry1Ah protein—a relatively higher temperature favored the degradation. The degradation rate of Cry1Ah protein was the fastest when the water content was 33 and 20% in the early and later stages, respectively. The soil types had a significant effect on the degradation of Cry1Ah protein. Moreover, soil sterilization slowed down the rate of protein degradation in both the early and later stages. In conclusion, the model *Y* = Y_0_ + ae^−b*X^ established in this study provided a more robust model for exploring and simulating the degradation of Bt protein in soil growing GM crops and overcame the shortcomings of the *Y* = ae^−b*X^ model. The findings of this study enriched the understanding of Bt protein degradation in soil ecosystems. They would be helpful for evaluating the environmental safety of GM crops.

## Introduction

Since the approval of FlavrSavr^®^ tomato, the first genetically modified (GM) food for human consumption, the market of GM foods has grown rapidly. The land area under cultivation of GM crops has been expanded to around ~190.4 million hectares, covering 12% of the global crop area. GM maize is the second-largest GM crop, covering ~60.9 million hectares and accounting for 32% of the global area under GM crops (ISAAA, 2019). China is the second-largest maize growing country globally, and GM maize has an important application potential in China. Moreover, the government policies of China have been committed to the research and development of GM crops, including GM maize, to facilitate their industrial use.

The crystalline (Cry) protein Cry1Ab derived from *Bacillus thuringiensis* (Bt) is mainly used to develop GM maize worldwide to manage insect pest infestations. It has been shown that the release of Bt proteins from Bt crops in soil ecosystems through root exudation (Morra, 1994), pollen, and stubble decomposition (Palm et al., 1994) affects the soil microbiota, functional groups, biodiversity, and soil ecological processes (Wang et al., 2005; Zeng et al., 2014; Shu et al., 2017; Liu et al., 2018). Therefore, several researchers have evaluated the degradation of Bt proteins, including Cry1Ac and Cry1Ab, in soil ecosystems (Zhang et al., 2007, 2015; Feng et al., 2011; Zaman et al., 2015). Similarly, the degradation of CP4-EPSPS protein in soil has also been reported (Zeng et al., 2018).

The first-order kinetics model (*Y* = ae^−b*X^) is widely used to assess the degradation dynamics of proteins/herbicides in soil (Zhang et al., 2015; Fang et al., 2016; Wang et al., 2016; Carretta et al., 2018; Zeng et al., 2018), because of its simple algorithm and high accuracy. However, in our data statistics process, this model demonstrates lower fitting accuracy in the presence of higher amounts of residual proteins, and it does not explain the influence of various factors on residual proteins in the later stage of exogenous protein degradation.

To expand the utilization of genetic resources and gain intellectual property protection, the *Cry1Ah* gene was cloned from the Bt8 strain by the Chinese Academy of Agricultural Sciences, and it has been used to develop GM maize GH5112E-117C (Han et al., 2016). Cry1Ah protein has a stronger insecticidal activity against many lepidopteron pests than Cry1Ac, Cry1Ab, and Cry1Ie proteins, but it has lower insecticidal activity against important economic insects, such as *Bombyxmori* (Xue et al., 2008; Chen et al., 2020). The productive test of GM maize GH5112E-117C, with potential industrial application prospects, has been completed, and it is now in the process of obtaining the Safety Certificate of Chinese GM Food Crops. However, studies on the degradation of Cry1Ah protein in soil have not been explored.

Therefore, in this study, we aimed to develop a more robust statistical model to assess the degradation dynamics of plant exogenous proteins in soil. The efficiency of the suggested model was investigated to assess the degradation of Cry1Ah protein released by GM maize GH5112E-117C. This method will provide a comprehensive understanding of Bt protein degradation in soil and facilitate assessment of the environmental safety of GM crops.

## Materials and Methods

### Plants

The seeds of GM maize GH5112E-117C were provided by Beijing Origin Seed Technology Inc.

The seeds were planted in the test field, and the leaves of the GM maize GH5112E-117C were randomly collected during the maize harvesting period and naturally air-dried. Afterward, the leaves were ground and sifted through a 1.0-mm sieve and stored in a freezer at −20°C for further analysis.

### Soil

Soil samples were collected from the 0–20 cm surface layer of maize fields growing non-GM maize in the main maize-producing areas of China. The soil types included two representative sites of China’s spring maize area in Gongzhuling and Beijing and two of China’s summer maize areas in Jinan and Zhengzhou. The basic physical and chemical properties of soil at these sites are shown in Table 1. The stones were removed from the soil, and the soil was naturally air-dried. Then, the samples were sifted using 1.0-mm sieves, homogenized, and kept for further analysis.

**Table 1:** The basic physical and chemical characters of the four soil types.

Sampling sites	Water content (%)	pH	Organic C (g·kg^−1^)	Nitrogen (g·kg^−1^)	Phosphorus (g·kg^−1^)	Potassium (g·kg^−1^)	Cation exchange capacity (g·kg^−1^)
Beijing	22.90	6.38	11.17	1.16	0.92	2.70	11.05
Gongzhuling	18.40	5.58	13.97	1.30	0.62	3.69	26.00
Jinan	20.00	6.77	10.17	1.09	0.61	4.54	16.73
Zhengzhou	15.00	6.24	2.79	0.50	0.51	3.91	6.27

### Estimation of the Effects of Temperature, Water Content, and Soil Types on Degradation of Cry1Ah Protein

For each centrifuge tube (2 ml), mixed corn leaves (0.1 g) and soil (0.9 g) were added. Afterward, to simulate the water content of the field soil (20, 33, and 50%), water (0.23, 0.45, and 0.9 g, respectively) was added to the centrifuge tubes. We perforated the cover of the centrifuge tubes with 2–3 holes (1 mm) and weighed each centrifuge tube every 1–2 d to supplement the evaporated water. The GM leaf–soil mixed samples were kept at three different temperatures (15°C, 25°C, and 35°C) in artificial climate chambers for 96 d. A total of 36 treatments (4 soil types × 3 water contents × 3 temperatures) were evaluated in this study.

Sampling was performed at 18 different time points in triplicate (0 h, 4 h, 8 h, 12 h, 16 h, 20 h, 24 h, 1.5d, 2d, 3d, 4d, 7d, 11d, 16d, 21d, 32d, 64d, and 96d). A total of 1944 (36 × 18 × 3) samples were investigated. After each sampling, the small holes on the centrifuge tubes were sealed with adhesive tape. The collected samples were stored in a refrigerator at −80°C for the subsequent analyses.

### Estimation of the Effects of Soil Sterilization on the Degradation of Cry1Ah Protein

To assess the effect of soil microbiota on the degradation of Cry1Ah protein, the soil samples obtained from Gongzhuling were sterilized thrice at 120°C (1 h each time) at an interval of 1d. The samples kept at 35°C with a simulated soil water content of 50%. The sampling was performed in three replicates at 18 different times, as described in previous section.

### Quantification of Bt Protein

The 0.1 g samples incubated in 10 ml centrifuge tubes were extracted using 8 ml of phosphate-buffered saline solution combined with 0.1% Tween-20 (PBST) provided with the kit, after which the extracts were clarified by centrifuging at 10000 × *g* for 4 min to achieve the best results. The supernatant was diluted to certain ratios (100:1, 30:1, 10:1 or 0) with PBST to suit the measuring range of the instrument. The presence of Bt proteins was assessed using an enzyme-linked immunosorbent assay (ELISA) kit [Bt-Cry1Ab/1Ac ELISA Kit (PSP 06200/0480, Agdia, USA)] following the manufacturer’s instructions. Quantification was corrected with different concentration of Cry1Ah protein solution. The purified Cry1Ah protein was provided by Institute of Biotechnology, Chinese Academy of Agricultural Sciences. After obtaining the measured value, the Bt protein concentration is calculated in combination with the water content.

### Statistical Analyses

#### Effect of Temperature, Water Content, and Soil Types on the Degradation of Cry1Ah Protein in Soil

The repeated measures module of IBM SPSS Statistics 23 software was used to perform ANOVA. The ELISA data of Cry1Ah protein under different conditions (temperature, water content, and soil types) were analyzed using the following operating procedure: Analyze/General Linear Models/Repeated Measures/Within-subject Factor Name: time/Number of levels:18/Define/select (h0 - d96) add Within-subjects variables time/select (Location, Water content, Temperature) add between subjects Factor/OK.

#### Effects of Soil Sterilization on the Degradation of Cry1Ah Protein

Since the sterilization experiment was not performed for all samples, the effect of sterilization on Cry1Ah protein degradation was analyzed separately. As described in previous section, ANOVA was performed using the ELISA data of Cry1Ah protein under different sterilization conditions. The operation procedure was the same as in previous section.

#### Model Fitting

The model of Cry1Ah protein degradation in soil was fitted by the exponential models (*Y* = ae^−b*X^ and *Y* = Y_0_ + ae^−b*X^) using the SigmaPlot version 12.0 (Systat Software, San Jose, CA). X denotes the degradation time (in days), *Y* is the amount of residues of Cry1Ah protein in the soil at time x, and the parameters Y_0_, a, and b are obtained after curve fitting.

The 50% degradation time (DT_50_) was calculated from the parameters fitted by the *Y* = ae^−b*X^ model using the following equation:


$${\text{DT}}_{50}={\text{Ln}}^{2}/\text{b}.$$

## Results

### ANOVA Analysis of the Degradation of Cry1Ah Protein in Soil Under Varying Temperature, Water Content, and Soil Types

The quantification data of Cry1Ah protein obtained under different conditions are shown in Supplementary Table S1. ANOVA results of the data are shown in Supplementary Table S2. In the multivariate test section, the analysis revealed significant differences (*p* < 0.01) between the average values of Cry1Ah protein residues obtained under different sampling times; it also showed significant interactions between the soil types, simulated water contents, temperatures, and sampling times. In the subject effects section, the results demonstrated significant variation in Cry1Ah protein residues with time, and the effect of soil types, simulated water content, and temperature significantly affected the changing trend of protein residues with time (*p* < 0.01). Additionally, the within-subject contracts section showed that the variation of Cry1Ah protein residues with time could be fitted via linear, quadric, cubic, and so on curves (*p* < 0.01). The between-subject effects section showed that the average value of Cry1Ah protein residue was different for different soil types, water content, and temperature (*p* < 0.01), and soil types, water content, and temperature demonstrated interaction effects (Supplementary Table S2).

### Degradation Curve of Cry1Ah Protein in Soil Under Different Conditions

#### Accuracy Comparison of Two Models

The curve simulation results showed that the degradation characteristics of Cry1Ah protein in soil complied with the *Y* = ae^−b*X^ and *Y =* Y_0_ + ae^−b*X^ models under all conditions. (All value of ps were less than 0.0001.) The fitting precision values (*R*^2^) of the *Y* = ae^−b*X^ model under different conditions varied from 0.8267 to 0.9944, with an average of 0.9434. However, the *Y =* Y_0_ + ae^−b*X^ model showed higher *R^2^* values (0.9152–0.9962 with an average is 0.9733). These results suggested a higher fitting accuracy of the *Y =* Y_0_ + ae^−b*X^ model than that of the *Y* = ae^−b*X^ model, especially when there are more residual proteins at the later stage of degradation under 15°C (Table 2).

**Table 2:** Degradation model of Cry1Ah protein in the soil collected from Gongzhuling (the results for the other soil types are shown in Supplementary Table S3).

Condition	Degradation model	*R* ^2^	Value of *p*	DT_50_(d)
15°C, 20%	*Y* = 336.25e^−0.046*t*^	0.9105	<0.0001	15.1
	*Y* = 83.58 + 266.69e^−0.0976*t*^	0.9727	<0.0001	
15°C, 33%	*Y =* 346.22e^−0.0661*t*^	0.9227	<0.0001	10.5
	*Y =* 74.37 + 286.73e^-0.1291*t*^	0.9756	<0.0001	
15°C, 50%	*Y* = 319.48e^−0.0378*t*^	0.8938	<0.0001	18.3
	*Y* = 87.33 + 246.25e^−0.0873*t*^	0.9640	<0.0001	
25°C, 20%	*Y* = 370.36e^−0.1199*t*^	0.9898	<0.0001	5.8
	*Y* = 22.09 + 352.16e^−0.1399*t*^	0.9939	<0.0001	
25°C, 33%	*Y* = 351.53e^−0.1146*t*^	0.9608	<0.0001	6.1
	*Y* = 44.53 + 319.43e^−0.1739*t*^	0.9811	<0.0001	
25°C, 50%	*Y* = 310.77e^−0.0747*t*^	0.9026	<0.0001	9.3
	*Y* = 51.36 + 274.35e^−0.1369*t*^	0.9215	<0.0001	
35°C, 20%	*Y* = 375.38e^−0.1861*t*^	0.9944	<0.0001	3.7
	*Y* = 15.92 + 362.61e^−0.2072*t*^	0.9962	<0.0001	
35°C, 33%	*Y* = 372.06e^−0.201*t*^	0.9750	<0.0001	3.5
	*Y* = 11.92 + 362.25e^−0.2168*t*^	0.9766	<0.0001	
35°C, 50%	*Y* = 347.45e^−0.0742*t*^	0.9916	<0.0001	9.3
	*Y* = 4.58 + 343.26e^−0.0762*t*^	0.9915	<0.0001	

**Figure 1:**
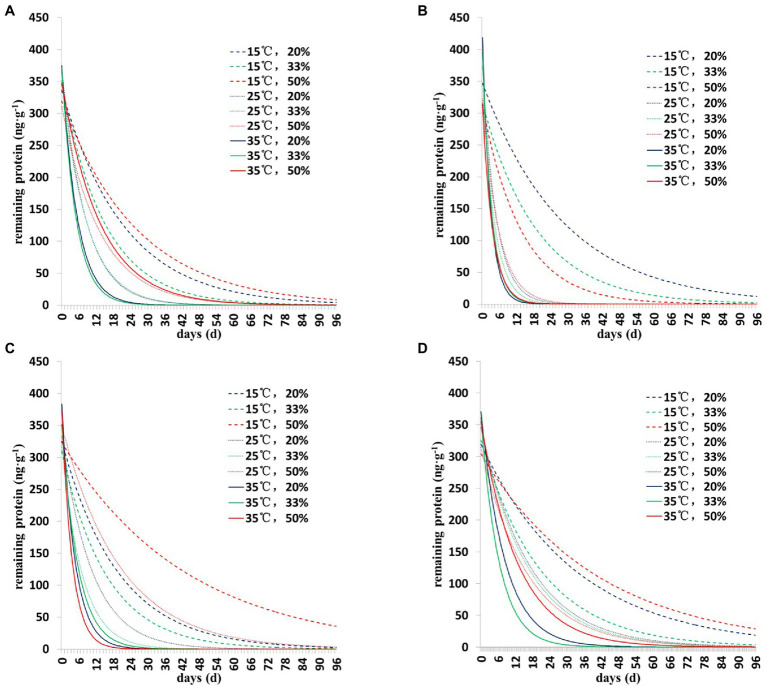
The degradation dynamics of Cry1Ah protein in the soil in four soil types under different environmental conditions. The data were calculated following degradation model *Y* = ae^−b*X^. **(A)** Gongzhuling, **(B)** Jinan, **(C)** Beijing, and **(D)** Zhengzhou; temperature (15°C, 25°C, and 35°C), water content (20, 33, and 50%).

**Figure 2:**
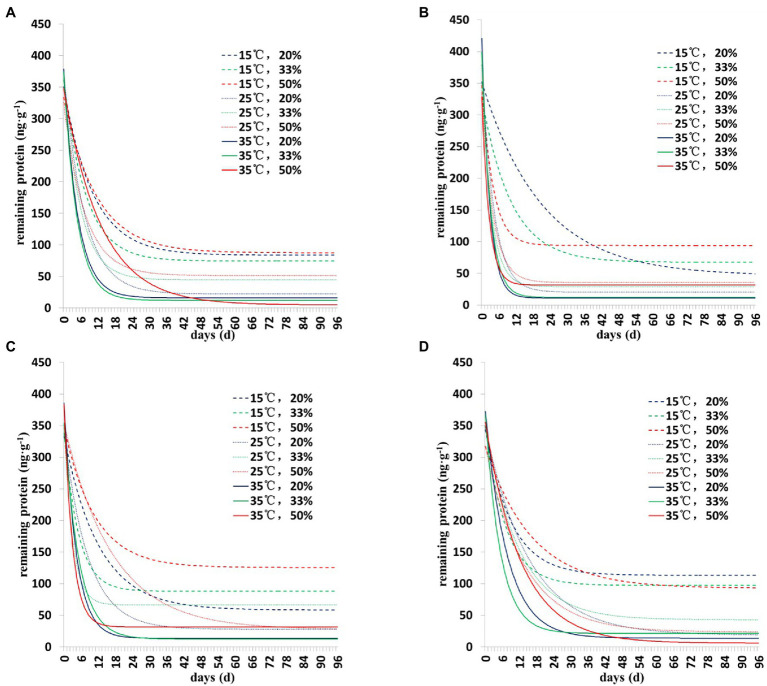
The degradation dynamics of Cry1Ah protein in the soil in four soil types under different environmental conditions. The data were calculated following degradation model *Y =* Y_0_ + ae^−b*X^. **(A)** Gongzhuling, **(B)** Jinan, **(C)** Beijing, and **(D)** Zhengzhou; temperature (15°C, 25°C, and 35°C), water content (20, 33, and 50%).

The degradation dynamics of Cry1Ah protein in soil were drawn based on the results of the two models (Figures 1, 2). Collectively, the results showed a similar pattern of degradation of Cry1Ah protein in soil under all conditions—a rapid degradation in the early stage followed by slow degradation in the middle and late stages. In particular, when the temperature was 15°C, the degradation rate was relatively slow, and there were more residual proteins in the late stage. These results were more clear when the curve was drawn using the *Y =* Y_0_ + ae^−b*X^ model (Figure 2).

#### Practical Meaning and Comparative Analysis of Parameter b

To analyze the effect of different conditions on the degradation of Cry1Ah protein, we analyzed the parameters of the two models under different conditions. The parameter b of both models has the same meaning and represents the degradation rate of Cry1Ah protein. It showed a strong correlation between the two models indicating that the parameter b in the *Y* = ae^−b*X^ model is similar to that of the *Y =* Y_0_ + ae^−b*X^ model. Since DT_50_ is directly derived from the parameter b of the *Y* = ae^−b*X^ model (DT_50_ = ln^2^/b), for convenience of description, we calculated DT_50_ to analyze the degradation of Cry1Ah protein in the early stage.

The average DT_50_ of Cry1Ah protein degradation in soil over all conditions was 9.8d. The results showed an inverse relation between DT_50_ and temperature—the average DT_50_ at 15°C, 25°C, and 35°C was 17.3d, 7.7d, and 4.3d, respectively. These results suggest that a higher temperature facilitates faster degradation. DT_50_ was the smallest (7.4d) at 33% water content, indicating the fastest degradation rate, whereas it was the slowest at 50% water content (DT_50_ = 12.4d). The average DT_50_ was 9.6d at 20% water content, indicating an intermediate degradation rate. The average DT_50_ in four soil types (Gongzhuling, Jinan, Beijing, and Zhengzhou) was 9.1, 6.6, 10.1, and 13.3d, respectively, indicating the fastest degradation rate in Jinan soil.

#### Practical Meaning and Comparative Analysis of Parameter Y_0_

Y_0_ is the minimum limit value of the model *Y =* Y_0_ + ae^−b*X^. The meaning of Y_0_ can be explained as the residual Cry1Ah protein that could not be degraded in the later stage. At 15°C, 25°C, and 35°C, the average values of Y_0_ were 85.7 ng·g^−1^, 34.2 ng·g^−1^, and 15.5 ng·g^−1^, respectively, indicating that the higher the temperature, the less the amount of residual protein. The average value of Y_0_ was 37.1 ng·g^−1^, 47.3 ng·g^−1^, and 50.1 ng·g^−1^ at 20, 33, and 50% water content, respectively, suggesting a positive relationship between the water content and residual protein. The average Y_0_ values in samples collected from Gongzhuling, Beijing, Jinan, and Zhengzhou were 43.9 ng·g^−1^, 38.6 ng·g^−1^, 50.2 ng·g^−1^, and 47.8 ng·g^−1^, respectively. The residual protein was the least in Jinan soil and the most in Beijing soil.

### Effects of Soil Sterilization on the Degradation of Cry1Ah Protein

The quantification data of Cry1Ah protein obtained under the sterilization condition are shown in Supplementary Table S4. The ANOVA results of the data are shown in Supplementary Table S5.

Sterilization had a significant impact on the residual amount of Cry1Ah protein, and the change in the amount of residual protein varied with time (Supplementary Table S5). The fitting accuracy of the model *Y =* Y_0_ + ae^−b*X^ (*R^2^* = 0.9935) was better than that of the model *Y* = ae^−b*X^ (*R^2^* = 0.9717) under sterilization conditions (Table 3).

**Table 3:** Degradation model of Cry1Ah protein in the soil (sterilized).

Condition	Degradation model	*R^2^*	Value of *p*	DT_50_ (d)
Sterilized	*Y* = 346.34e^−0.059*t*^	0.9717	<0.0001	11.75
	*Y* = 48.82 + 302.95e^−0.0827*t*^	0.9935	<0.0001	
Nonsterilized	*Y* = 347.45e^−0.0742*t*^	0.9916	<0.0001	9.34
	*Y* = 4.58 + 343.26e^−0.0762*t*^	0.9915	<0.0001	

**Figure 3:**
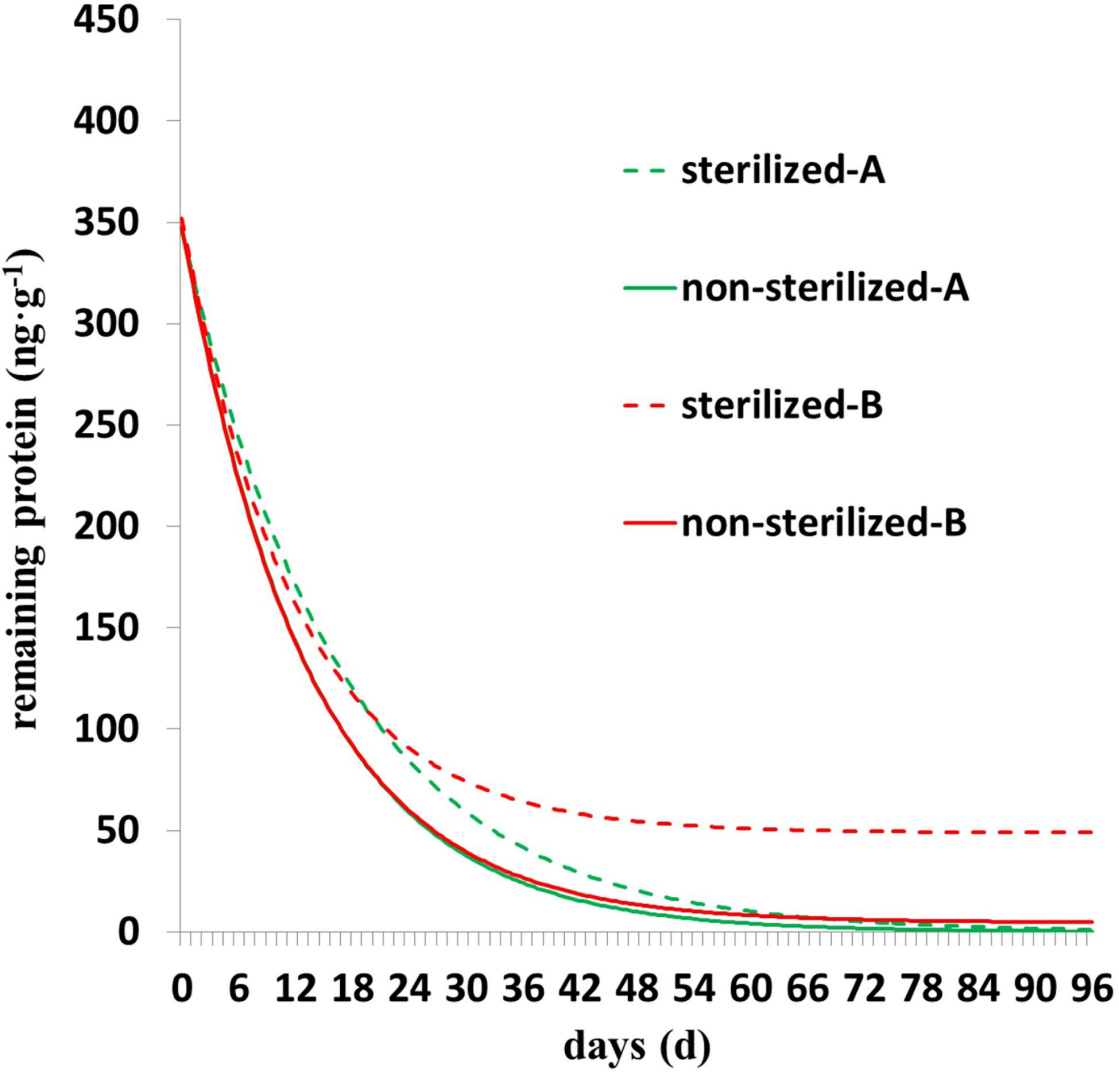
The degradation dynamics of Cry1Ah protein in nonsterilized and sterilized soils. A (degradation model *Y* = ae^−b*X^), B (degradation model *Y =* Y_0_ + ae^−b*X^).

As shown in Figure 3, the degradation of Cry1Ah protein is characterized by rapid degradation in the early stage and slow degradation in the middle and late stages. The residual protein in the sterilized soil was higher than that in the nonsterilized soil, and the trend was clearer in the *Y =* Y_0_ + ae^−b*X^ model (Figure 3). In nonsterilized soil, there was less residual protein in the later stage, the parameter Y_0_ of *Y =* Y_0_ + ae^−b*X^ model was close to zero, and parameter b was close to the parameter b of *Y* = ae^−b*X^; in addition, the fitting accuracy (Table 3) and fitting curves (Figure 3) of the two models were similar.

## Discussion

### Repeated Measures ANOVA

Repeated measure designs are designs in which the same subject is measured more than once at different times or environments, and they are used to analyze the changing trend of the observations and related influencing factors. However, several researchers have used multi-way ANOVA (time was regarded as a factor equal to temperature and water content) to study the degradation of Bt protein or to directly compare different factors at a single time point without considering the correlation of the observations at different time points or the changing trend of the observations with time (Bai et al., 2005; Zhang et al., 2007, 2015; Feng et al., 2011; Zeng et al., 2018).

The repeated measures ANOVA analysis in this paper not only proved that the amount of Bt protein residues changed consistently with time but also proved that location, water content, temperature, and sterilization had a significant impact on the degradation of Bt proteins with time and laid a foundation for the model fitting and statistical description of Bt protein degradation in soil.

### Whole and Part in Statistical Analysis

In this study, we replaced the broken line graph reported in earlier studies with the fitting curve (Feng and Simpson, 2009; Feng et al., 2011; Slade and Riutta, 2012; Zeng et al., 2018), which intuitively shows the degradation of Bt protein with time and the influence of different factors on the degradation trend. Error is inevitable in data statistics, and every measured value has a certain deviation from the real value. However, the results obtained by model fitting are closer to the real value than the measured value. Furthermore, the degradation of Bt protein with time is a continuous process; therefore, the results should show continuous change.

ANOVA is based on the principle of the whole before part. In other words, if the result of the ANOVA is not significant in general, it is not necessary to analyze every time point. On the contrary, if the ANOVA gets significant results in general, each time point should be analyzed, and the time points with differences should be determined. However, even if there is no significant difference in the data at each time point, we think that this factor has a significant impact on the observed variables. This is because the ANOVA result, in general, is the sum of all samples and is concluded by examining all the data. For example, Feng et al. (2011) conducted ANOVA on the data at each time point and concluded that soil pH value and water content had no significant effect on the degradation of Cry1Ab (Feng et al., 2011); however, we observed contrasting results, and found that the degradation at high pH was smaller than that at low pH at most time points. Some other studies had encountered similar problems (Bai et al., 2005; Zhang et al., 2007, 2015; Zeng et al., 2018). Therefore, to get a more scientific conclusion, we suggest that repeated measures ANOVA should be adopted.

### Model Fitting

Repeated measures ANOVA showed that the variation of Cry1Ah protein residues with time could be fitted by linear, quadric, and cubic curves (Supplementary Table S3). In previous studies, linear, asymmetric, quadratic, power functions, exponential model, and shift log model have been used to study the degradation of various substances in soil (Wider and Lang, 1982; Sims and Holden, 1996; Herman et al., 2002a,b; Feng et al., 2011; Slade and Riutta, 2012). However, in recent years, the first-order kinetics model (*Y* = ae^−b*X^) has been adopted by most studies (Zhang et al., 2015; Fang et al., 2016; Wang et al., 2016; Carretta et al., 2018; Zeng et al., 2018), probably because of its simple calculation of methods and higher fitting precision value compared to that of the other models, the other models are either less accurate or more complex, and whose parameters are difficult to explain.

The *Y =* Y_0_ + ae^−b*X^ model established and applied in this study shows higher accuracy than the *Y* = ae^−b*X^ model, with an average *R^2^* difference of 0.03. Y_0_ is the minimum limit value of the model *Y =* Y_0_ + ae^−b*X^ representing the residual Cry1Ah protein that cannot be degraded in the later stage. *Y =* Y_0_ + ae^−b*X^ showed higher accuracy, mainly with more residual proteins in the later stage, whereas with lower amounts of residual protein in the later stage, the parameter Y_0_ is close to zero, and the parameter b and fitting accuracy of the two models tend to be the same. Similarly, three of the four curves in Figure 3 almost overlap, and the difference between the curves fitted by *Y* = ae^−b*X^ between the two groups of data is not obvious, the curve fitted by *Y =* Y_0_ + ae^−b*X^ can clearly see the difference between the two groups of data, and the curve fitted by *Y =* Y_0_ + ae^−b*X^ almost coincides with the measured value under sterilization conditions (*R^2^* = 0.9935). Overall, the model *Y =* Y_0_ + ae^−b*X^ resolves the problem that the fitting accuracy of *Y* = ae^−b*X^ is lower when the residual protein is higher in a later stage, and it can completely replace *Y* = ae^−b*X^. The model *Y =* Y_0_ + ae^−b*X^ unraveled precise estimation of the influence of various factors on the degradation of Cry1Ah protein in soil. The model directly describes the effect of different gradients of different factors on the degradation of Cry1Ah protein in soil. Moreover, based on the comparison of the range of different parameters, it explains that the factors affecting the early stage degradation of Cry1Ah protein are temperature > soil types > water content > sterilization (13.0d > 6.7d > 5.0d > 2.4d), and those in the later stage are temperature > sterilization > water content > soil types (70.2 ng·g^−1^ > 44.2 ng·g^−1^ > 13.0 ng·g^−1^ > 11.5 ng·g^−1^).

### Regulation of Bt Protein Degradation in Soils

Several studies have shown that the residual proteins released from the GM crops follow a typical pattern of a rapid rate of degradation in the early stage followed by a slow rate in the middle and late stages (Bai et al., 2006; Feng et al., 2011; Zhang et al., 2015; Zeng et al., 2018). Accordingly, our results also demonstrated similar patterns of degradation dynamics of Cry1Ah protein in soil. Feng et al. (2011) demonstrated that soil temperature had significant effects on the degradation of Cry1Ab protein released from two kinds of GM maize straw, with a higher degradation rate at a higher temperature; however, soil water content and pH had no effects (Feng et al., 2011). Zeng et al. (2018) reported that the soil temperature and sterilization significantly affected the degradation of CP4-EPSPS protein released from maize leaves, whereas the soil water content and pH did not reveal any significant effect (Zeng et al., 2018). It has also been shown that the Cry1Ac protein in the Bt cotton leaves and buds was degraded most rapidly in the early stage at 35°C with 70% soil water holding capacity, and soil temperature was a major factor influencing the degradation of Cry1Ac protein from Bt cotton residues (Zhang et al., 2015). Another study showed that flooding could accelerate the decomposition of rice tissues and thus enhance the degradation of Cry1Ab protein, and microbes in soils had positive effects on the Cry1Ab degradation (Bai et al., 2005). It has also been shown that the degradation rate of Cry1Ab was influenced by the soil water content, pH, and temperature, of which the effects of soil pH and temperature were relatively more pronounced (Bai et al., 2006). Collectively, it can be inferred that temperature has the greatest impact on the degradation of Bt protein in soil. The higher the temperature, the faster the degradation rate, and the less the residual protein in the late stage. This regular pattern is not only suitable for the degradation of Bt protein in soil but also suitable for the degradation of other substances (Feng and Simpson, 2009; Fang et al., 2016; Wang et al., 2016; Carretta et al., 2018; Velásquez-Castillo et al., 2020), including total organic matter in the soil (Bosatta and Agren, 1999; Wettersted et al., 2010).

Previous studies have demonstrated inconsistent results for other factors (water content, pH value, and sterilization). Moreover, some results were not statistically significant (Palm et al., 1996; Feng et al., 2011; Zhang et al., 2015; Zeng et al., 2018), which could be due to the insufficient sample size of the experimental design (few repetitions or Latin square design) and the insufficient gradient settings. However, the impact of these factors on the degradation of Bt protein in the soil cannot be ignored. For example, the factors that change in a curve only take the data that have a greater or smaller impact on the degradation, there is not enough variation in the observed values of variables, and the statistical model cannot capture the changes of variables. Moreover, it does not consider the continuous change of time and does not carry out ANOVA on the whole data, but independently carries out ANOVA on the data of each time point to summarize the whole.

## Conclusion

In this study, a new model (*Y =* Y_0_ + ae^−b*X^) is established to study the degradation of Bt protein in soil. The accuracy of the new regression model is higher, and through the analysis of parameters b and Y_0_, the study provides a better understanding of the degradation of Bt protein in the soil. The results show that Bt protein in soil degrades rapidly in the early stage and slowly in the middle and late stages. The factors affecting its early stage degradation are temperature > soil types > water content > sterilization, whereas those affecting the residues in the later stage are temperature > sterilization > water content > soil types. Collectively, these findings show that a relatively higher temperature facilitates a faster degradation rate in the early stage and less residual protein in the later stage, suggesting that the Cry1Ah protein might not accumulate in soil under appropriate environmental conditions. However, the precise effects of environmental factors in natural field conditions should be ascertained by further studies to realize the environmental impacts of GM maize.

## Data Availability Statement

The original contributions presented in the study are included in the article/Supplementary Material, further inquiries can be directed to the corresponding authors.

## Author Contributions

XS and LL brought the idea and designed the trial. ZJ and LZ interpreted the results and wrote the paper. ZJ, LZ, BW, JY, FW, and DW performed the experiments. All authors contributed to the manuscript and approved the final version.

## Funding

This study was supported by the National Natural Science Foundation of China (grant number 32071546).

## Conflict of Interest

The authors declare that the research was conducted in the absence of any commercial or financial relationships that could be construed as a potential conflict of interest.

## Publisher’s Note

All claims expressed in this article are solely those of the authors and do not necessarily represent those of their affiliated organizations, or those of the publisher, the editors and the reviewers. Any product that may be evaluated in this article, or claim that may be made by its manufacturer, is not guaranteed or endorsed by the publisher.

## Supplementary Material

Supplementary Table S1 The ELISA results on the degradation of Cry1Ah protein under different conditions of temperature, water content, and soil types.

Supplementary Table S2 The ANOVA of the degradation data of Cry1Ah protein under different conditions of temperature, water content, and soil types.

Supplementary Table S3 Degradation model of Cry1Ah protein in the soil.

Supplementary Table S4 The ELISA results on the degradation of Cry1Ah protein under soil sterilization and non-sterilization conditions.

Supplementary Table S5 The ANOVA of the degradation data of Cry1Ah protein under soil sterilization and non-sterilization conditions.
