# Supplementary material for: Construction of a Novel Degradation Model of Bacillus thuringiensis Protein in Soil and Its Application in Estimation of the Degradation Dynamics of Bt-Cry1Ah Protein
Source: Front Plant Sci. 2022 Apr 13;13:875020. doi: 10.3389/fpls.2022.875020 (PMC9043894; doi:10.3389/fpls.2022.875020)
Supplement: Supplementary Table S2 — The ANOVA of the degradation data of Cry1Ah protein under different conditions of temperature, water content, and soil types. [file Table_2.DOCX]

Table S3. Degradation model of Cry1Ah protein in the soil（Temperature，Humidity，Region）.

| Condition | Degradation model | *R^2^* | *P* | DT_50_（d） |
| --- | --- | --- | --- | --- |
| G,15℃,20% | *Y*=336.25e^-0.046^*^t^* | 0.9105 | <0.0001 | 15.1 |
|  | *Y*=83.58+266.69e^-0.0976^*^t^* | 0.9727 | <0.0001 |  |
| G,15℃,33% | *Y=*346.22e^-0.0661^*^t^* | 0.9227 | <0.0001 | 10.5 |
|  | *Y=*74.37+286.73e^-0.1291^*^t^* | 0.9756 | <0.0001 |  |
| G,15℃,50% | *Y*=319.48e^-0.0378^*^t^* | 0.8938 | <0.0001 | 18.3 |
|  | *Y*=87.33+246.25e^-0.0873^*^t^* | 0.964 | <0.0001 |  |
| G,25℃,20% | *Y*=370.36e^-0.1199^*^t^* | 0.9898 | <0.0001 | 5.8 |
|  | *Y*=22.09+352.16e^-0.1399^*^t^* | 0.9939 | <0.0001 |  |
| G,25℃,33% | *Y*=351.53e^-0.1146^*^t^* | 0.9608 | <0.0001 | 6.1 |
|  | *Y*=44.53+319.43e^-0.1739^*^t^* | 0.9811 | <0.0001 |  |
| G,25℃,50% | *Y*=310.77e^-0.0747^*^t^* | 0.9026 | <0.0001 | 9.3 |
|  | *Y*=51.36+274.35e^-0.1369^*^t^* | 0.9215 | <0.0001 |  |
| G,35℃,20% | *Y*=375.38e^-0.1861^*^t^* | 0.9944 | <0.0001 | 3.7 |
|  | *Y*=15.92+362.61e^-0.2072^*^t^* | 0.9962 | <0.0001 |  |
| G,35℃,33% | *Y*=372.06e^-0.201^*^t^* | 0.975 | <0.0001 | 3.5 |
|  | *Y*=11.92+362.25e^-0.2168^*^t^* | 0.9766 | <0.0001 |  |
| G,35℃,50% | *Y*=347.45e^-0.0742^*^t^* | 0.9916 | <0.0001 | 9.3 |
|  | *Y*=4.58+343.26e^-0.0762^*^t^* | 0.9915 | <0.0001 |  |
| J,15℃,20% | *Y*=347.58e^-0.0352^*^t^* | 0.969 | <0.0001 | 19.7 |
|  | *Y*=46.25+305.08e^-0.0473^*^t^* | 0.9822 | <0.0001 |  |
| J,15℃,33% | *Y*=315.86e^-0.0525^*^t^* | 0.9102 | <0.0001 | 13.2 |
|  | *Y*=67.65+258.88e^-0.0972^*^t^* | 0.9566 | <0.0001 |  |
| J,15℃,50% | *Y*=311.54e^-0.0731^*^t^* | 0.8337 | <0.0001 | 9.5 |
|  | *Y*=94.11+252.22e^-0.2545^*^t^* | 0.9712 | <0.0001 |  |
| J,25℃,20% | *Y*=374.45e^-0.2083^*^t^* | 0.9874 | <0.0001 | 3.3 |
|  | *Y*=20.39+358.38e^-0.2393^*^t^* | 0.992 | <0.0001 |  |
| J,25℃,33% | *Y*=345.51e^-0.2384^*^t^* | 0.9746 | <0.0001 | 2.9 |
|  | *Y*=29.02+323.59e^-0.2985^*^t^* | 0.9875 | <0.0001 |  |
| J,25℃,50% | *Y*=322.2e^-0.1792^*^t^* | 0.9628 | <0.0001 | 3.9 |
|  | *Y*=35.62+298.70e^-0.2582^*^t^* | 0.9848 | <0.0001 |  |
| J,35℃,20% | *Y*=419.11e^-0.3531^*^t^* | 0.9869 | <0.0001 | 2 |
|  | *Y*=10.95+409.74e^-0.3727^*^t^* | 0.988 | <0.0001 |  |
| J,35℃,33% | *Y*=405.75e^-0.3016^*^t^* | 0.9741 | <0.0001 | 2.3 |
|  | *Y*=12.17+386.52e^-0.3338^*^t^* | 0.9888 | <0.0001 |  |
| J,35℃,50% | *Y*=314.12e^-0.2788^*^t^* | 0.9289 | <0.0001 | 2.5 |
|  | *Y*=31.50+296.97e^-0.3963^*^t^* | 0.9502 | <0.0001 |  |
| B,15℃,20% | *Y*=325.12e^-0.0506^*^t^* | 0.9504 | <0.0001 | 13.7 |
|  | *Y*=58.29+274.61e^-0.0815^*^t^* | 0.9815 | <0.0001 |  |
| B,15℃,33% | *Y*=309.34e^-0.0634^*^t^* | 0.8705 | <0.0001 | 10.9 |
|  | *Y*=88.12+250.03e^-0.1997^*^t^* | 0.9542 | <0.0001 |  |
| B,15℃,50% | *Y*=323.42e^-0.0229^*^t^* | 0.8267 | <0.0001 | 30.3 |
|  | *Y*=125.57+221.06e^-0.0926^*^t^* | 0.93 | <0.0001 |  |
| B,25℃,20% | *Y*=333.48e^-0.0947^*^t^* | 0.9657 | <0.0001 | 7.3 |
|  | *Y*=27.91+310.35e^-0.1184^*^t^* | 0.9728 | <0.0001 |  |
| B,25℃,33% | *Y*=318.73e^-0.1402^*^t^* | 0.89 | <0.0001 | 4.9 |
|  | *Y*=66.30+285.56e^-0.3378^*^t^* | 0.9705 | <0.0001 |  |
| B,25℃,50% | *Y*=349.49e^-0.0491^*^t^* | 0.977 | <0.0001 | 14.1 |
|  | *Y*=27.73+324.56e^-0.0588^*^t^* | 0.9817 | <0.0001 |  |
| B,35℃,20% | *Y*=383.51e^-0.2191^*^t^* | 0.9944 | <0.0001 | 3.2 |
|  | *Y*=13.71+372.03e^-0.2376^*^t^* | 0.9959 | <0.0001 |  |
| B,35℃,33% | *Y*=351.83e^-0.1763^*^t^* | 0.9854 | <0.0001 | 3.9 |
|  | *Y*=12.48+341.34e^-0.1914^*^t^* | 0.9867 | <0.0001 |  |
| B,35℃,50% | *Y*=375.89e^-0.2737^*^t^* | 0.9742 | <0.0001 | 2.5 |
|  | *Y*=31.48+351.81e^-0.3391^*^t^* | 0.9899 | <0.0001 |  |
| Z,15℃,20% | *Y*=319.29e^-0.0295^*^t^* | 0.8384 | <0.0001 | 23.5 |
|  | *Y*=113.05+230.27e^-0.1098^*^t^* | 0.9152 | <0.0001 |  |
| Z,15℃,33% | *Y*=325.79e^-0.0473^*^t^* | 0.87 | <0.0001 | 14.7 |
|  | *Y*=97.32+251.43e^-0.1385^*^t^* | 0.956 | <0.0001 |  |
| Z,15℃,50% | *Y*=304.14e^-0.0246^*^t^* | 0.8937 | <0.0001 | 28.2 |
|  | *Y*=92.84+224.14e^-0.0621^*^t^* | 0.9515 | <0.0001 |  |
| Z,25℃,20% | *Y*=345.48e^-0.0582^*^t^* | 0.9717 | <0.0001 | 11.9 |
|  | *Y*=18.82+328.46e^-0.0656^*^t^* | 0.974 | <0.0001 |  |
| Z,25℃,33% | *Y*=343.87e^-0.0612^*^t^* | 0.9543 | <0.0001 | 11.3 |
|  | *Y*=42.92+307.10e^-0.0851^*^t^* | 0.9674 | <0.0001 |  |
| Z,25℃,50% | *Y*=314.28e^-0.0616^*^t^* | 0.9658 | <0.0001 | 11.3 |
|  | *Y*=23.70+293.55e^-0.0741^*^t^* | 0.9702 | <0.0001 |  |
| Z,35℃,20% | *Y*=370.69e^-0.1193^*^t^* | 0.9931 | <0.0001 | 5.8 |
|  | *Y*=13.99+358.79e^-0.1307^*^t^* | 0.9939 | <0.0001 |  |
| Z,35℃,33% | *Y*=361.74e^-0.1564^*^t^* | 0.9834 | <0.0001 | 4.4 |
|  | *Y*=21.37+345.85e^-0.1862^*^t^* | 0.9873 | <0.0001 |  |
| Z,35℃,50% | *Y*=354.94e^-0.0767^*^t^* | 0.987 | <0.0001 | 9 |
|  | *Y*=6.08+349.37e^-0.0795^*^t^* | 0.9868 | <0.0001 |  |

Note：G，Gongzhuling；J，Jinan；B，Beijing； Z，Zhengzhou.
