# Supplementary material for: Construction of a Novel Degradation Model of Bacillus thuringiensis Protein in Soil and Its Application in Estimation of the Degradation Dynamics of Bt-Cry1Ah Protein
Source: Front Plant Sci. 2022 Apr 13;13:875020. doi: 10.3389/fpls.2022.875020 (PMC9043894; doi:10.3389/fpls.2022.875020)
Supplement: Supplementary Table S4 — The ELISA results on the degradation of Cry1Ah protein under soil sterilization and non-sterilization conditions. [file Data_Sheet_1.PDF]

```
GLM h0 h4 h8 h12 h16 h20 h24 d1.5 d2 d3 d4 d7 d11 d16 d21 d32 d64 d96 BY Location Humidity
  Temperature
 /WSFACTOR= time 18 Polynomial
 /METHOD=SSTYPE(3)
 /CRITERIA=ALPHA(.05)
 /WSDESIGN= time
 /DESIGN=Location Humidity Temperature Location*Humidity Location*Temperature Humidity*Temperature
  Location*Humidity*Temperature.
```

General Linear Model

Notes

Output Created		09-JUL-2020 15:01:00
Comments		
Input	Data	C:\360CloudEnterprise\Cache\557795417\14829270102172675\周蕾\数据\原始数据.sav
	Active Dataset	DataSet1
	Filter	<none>
	Weight	<none>
	Split File	<none>
	N of Rows in Working Data File	108
Missing Value Handling	Definition of Missing	User-defined missing values are treated as missing.
	Cases Used	Statistics are based on all cases with valid data for all variables in the model.
Syntax		GLM h0 h4 h8 h12 h16 h20 h24 d1.5 d2 d3 d4 d7 d11 d16 d21 d32 d64 d96 BY Location Humidity Temperature /WSFACTOR=time 18 Polynomial /METHOD=SSTYPE(3) /CRITERIA=ALPHA(.05) /WSDESIGN=time /DESIGN=Location Humidity Temperature Location*Humidity Location*Temperature Humidity*Temperature Location*Humidity*Temperature.
Resources	Processor Time	00:00:00.05
	Elapsed Time	00:00:00.10

[DataSet1] C:\360CloudEnterprise\Cache\557795417\14829270102172675\周蕾\数据\原始数据.sav

Within-Subjects Factors

Measure: MEASURE_1

time	Dependent Variable
1	h0
2	h4
3	h8
4	h12
5	h16
6	h20
7	h24
8	d1.5
9	d2
10	d3
11	d4
12	d7
13	d11
14	d16
15	d21
16	d32
17	d64
18	d96

Between-Subjects Factors

		N
Location	Beijing	27
	Gongzhuling	27
	Jinan	27
	Zhengzhou	27
Humidity	20	36
	33	36
	50	36
Temperature	15	36
	25	36
	35	36

Multivariate Tests[a]

Effect		Value	F	Hypothesis df	Error df	Sig.
time	Pillai's Trace	1.000	36920.729[b]	17.000	56.000	.000
	Wilks' Lambda	.000	36920.729[b]	17.000	56.000	.000
	Hotelling's Trace	11208.078	36920.729[b]	17.000	56.000	.000
	Roy's Largest Root	11208.078	36920.729[b]	17.000	56.000	.000
time * Location	Pillai's Trace	2.489	16.624	51.000	174.000	.000
	Wilks' Lambda	.001	30.519	51.000	167.527	.000
	Hotelling's Trace	57.931	62.096	51.000	164.000	.000
	Roy's Largest Root	49.972	170.494[c]	17.000	58.000	.000
time * Humidity	Pillai's Trace	1.872	48.845	34.000	114.000	.000
	Wilks' Lambda	.003	59.622[b]	34.000	112.000	.000
	Hotelling's Trace	44.865	72.576	34.000	110.000	.000
	Roy's Largest Root	36.008	120.732[c]	17.000	57.000	.000
time * Temperature	Pillai's Trace	1.921	81.681	34.000	114.000	.000
	Wilks' Lambda	.000	157.995[b]	34.000	112.000	.000
	Hotelling's Trace	187.058	302.593	34.000	110.000	.000
	Roy's Largest Root	174.389	584.715[c]	17.000	57.000	.000
time * Location * Humidity	Pillai's Trace	4.108	7.788	102.000	366.000	.000
	Wilks' Lambda	.000	11.575	102.000	326.258	.000
	Hotelling's Trace	31.545	16.803	102.000	326.000	.000
	Roy's Largest Root	18.354	65.860[c]	17.000	61.000	.000
time * Location * Temperature	Pillai's Trace	3.559	5.233	102.000	366.000	.000
	Wilks' Lambda	.000	11.422	102.000	326.258	.000
	Hotelling's Trace	53.246	28.363	102.000	326.000	.000
	Roy's Largest Root	38.914	139.634[c]	17.000	61.000	.000
time * Humidity * Temperature	Pillai's Trace	3.296	16.257	68.000	236.000	.000
	Wilks' Lambda	.000	20.422	68.000	222.078	.000
	Hotelling's Trace	33.290	26.681	68.000	218.000	.000
	Roy's Largest Root	21.422	74.347[c]	17.000	59.000	.000
time * Location * Humidity * Temperature	Pillai's Trace	6.095	4.068	204.000	804.000	.000
	Wilks' Lambda	.000	8.517	204.000	579.356	.000
	Hotelling's Trace	72.757	19.319	204.000	650.000	.000
	Roy's Largest Root	36.143	142.445[c]	17.000	67.000	.000

a. Design: Intercept + Location + Humidity + Temperature + Location * Humidity + Location * Temperature + Humidity * Temperature + Location * Humidity * Temperature
Within Subjects Design: time

b. Exact statistic

c. The statistic is an upper bound on F that yields a lower bound on the significance level.

Mauchly's Test of Sphericity[a]

Measure: MEASURE_1

Within Subjects Effect	Mauchly's W	Approx. Chi-Square	df	Sig.	Epsilon[b] Greenhouse-Geisser	Huynh-Feldt	Lower-bound
time	.008	321.627	152	.000	.617	1.000	.059

Tests the null hypothesis that the error covariance matrix of the orthonormalized transformed dependent variables is proportional to an identity matrix.

a. Design: Intercept + Location + Humidity + Temperature + Location * Humidity + Location * Temperature + Humidity * Temperature + Location * Humidity * Temperature
Within Subjects Design: time

b. May be used to adjust the degrees of freedom for the averaged tests of significance. Corrected tests are displayed in the Tests of Within-Subjects Effects table.

Tests of Within-Subjects Effects

Measure: MEASURE_1

Source		Type III Sum of Squares	df	Mean Square	F	Sig.
time	Sphericity Assumed	25792265.17	17	1517192.069	26100.607	.000
	Greenhouse-Geisser	25792265.17	10.493	2457996.128	26100.607	.000
	Huynh-Feldt	25792265.17	17.000	1517192.069	26100.607	.000
	Lower-bound	25792265.17	1.000	25792265.17	26100.607	.000
time * Location	Sphericity Assumed	167461.196	51	3283.553	56.488	.000
	Greenhouse-Geisser	167461.196	31.480	5319.669	56.488	.000
	Huynh-Feldt	167461.196	51.000	3283.553	56.488	.000
	Lower-bound	167461.196	3.000	55820.399	56.488	.000
time * Humidity	Sphericity Assumed	143602.911	34	4223.615	72.660	.000
	Greenhouse-Geisser	143602.911	20.986	6842.660	72.660	.000
	Huynh-Feldt	143602.911	34.000	4223.615	72.660	.000
	Lower-bound	143602.911	2.000	71801.455	72.660	.000
time * Temperature	Sphericity Assumed	681600.804	34	20047.082	344.875	.000
	Greenhouse-Geisser	681600.804	20.986	32478.189	344.875	.000
	Huynh-Feldt	681600.804	34.000	20047.082	344.875	.000
	Lower-bound	681600.804	2.000	340800.402	344.875	.000
time * Location * Humidity	Sphericity Assumed	70494.769	102	691.125	11.890	.000
	Greenhouse-Geisser	70494.769	62.959	1119.689	11.890	.000
	Huynh-Feldt	70494.769	102.000	691.125	11.890	.000
	Lower-bound	70494.769	6.000	11749.128	11.890	.000
time * Location * Temperature	Sphericity Assumed	180182.413	102	1766.494	30.389	.000
	Greenhouse-Geisser	180182.413	62.959	2861.889	30.389	.000
	Huynh-Feldt	180182.413	102.000	1766.494	30.389	.000
	Lower-bound	180182.413	6.000	30030.402	30.389	.000
time * Humidity * Temperature	Sphericity Assumed	89901.480	68	1322.081	22.744	.000
	Greenhouse-Geisser	89901.480	41.973	2141.897	22.744	.000
	Huynh-Feldt	89901.480	68.000	1322.081	22.744	.000
	Lower-bound	89901.480	4.000	22475.370	22.744	.000
time * Location * Humidity * Temperature	Sphericity Assumed	213508.454	204	1046.610	18.005	.000
	Greenhouse-Geisser	213508.454	125.918	1695.608	18.005	.000
	Huynh-Feldt	213508.454	204.000	1046.610	18.005	.000
	Lower-bound	213508.454	12.000	17792.371	18.005	.000
Error(time)	Sphericity Assumed	71149.422	1224	58.129		
	Greenhouse-Geisser	71149.422	755.511	94.174		

Tests of Within-Subjects Effects

Measure: MEASURE_1

Source		Type III Sum of Squares	df	Mean Square	F	Sig.
	Huynh-Feldt	71149.422	1224.000	58.129		
	Lower-bound	71149.422	72.000	988.186		

Tests of Within-Subjects Contrasts

Measure: MEASURE_1

Source	time	Type III Sum of Squares	df	Mean Square	F	Sig.
time	Linear	25194715.22	1	25194715.22	374856.496	.000
	Quadratic	321328.532	1	321328.532	5583.523	.000
	Cubic	116151.365	1	116151.365	2070.446	.000
	Order 4	122882.297	1	122882.297	2044.235	.000
	Order 5	10876.359	1	10876.359	157.785	.000
	Order 6	6583.795	1	6583.795	146.892	.000
	Order 7	2821.547	1	2821.547	55.691	.000
	Order 8	16.085	1	16.085	.283	.597
	Order 9	252.814	1	252.814	5.129	.027
	Order 10	2444.040	1	2444.040	34.304	.000
	Order 11	1682.905	1	1682.905	31.004	.000
	Order 12	7857.693	1	7857.693	132.398	.000
	Order 13	2876.878	1	2876.878	56.044	.000
	Order 14	880.160	1	880.160	15.949	.000
	Order 15	121.905	1	121.905	2.231	.140
	Order 16	671.758	1	671.758	10.630	.002
	Order 17	101.814	1	101.814	1.512	.223
time * Location	Linear	25044.558	3	8348.186	124.207	.000
	Quadratic	82199.287	3	27399.762	476.108	.000
	Cubic	32309.169	3	10769.723	191.975	.000
	Order 4	6439.923	3	2146.641	35.711	.000
	Order 5	7342.184	3	2447.395	35.505	.000
	Order 6	1649.208	3	549.736	12.265	.000
	Order 7	1590.403	3	530.134	10.464	.000
	Order 8	927.586	3	309.195	5.431	.002
	Order 9	1462.913	3	487.638	9.893	.000
	Order 10	2535.167	3	845.056	11.861	.000
	Order 11	474.473	3	158.158	2.914	.040
	Order 12	2737.986	3	912.662	15.378	.000
	Order 13	857.075	3	285.692	5.566	.002
	Order 14	175.898	3	58.633	1.062	.370
	Order 15	918.823	3	306.274	5.606	.002
	Order 16	724.380	3	241.460	3.821	.013
	Order 17	72.163	3	24.054	.357	.784
time * Humidity	Linear	71597.582	2	35798.791	532.628	.000
	Quadratic	17831.389	2	8915.695	154.922	.000
	Cubic	37667.303	2	18833.652	335.718	.000
	Order 4	2679.002	2	1339.501	22.284	.000

Tests of Within-Subjects Contrasts

Measure: MEASURE_1

Source	time	Type III Sum of Squares	df	Mean Square	F	Sig.
	Order 5	6859.706	2	3429.853	49.757	.000
	Order 6	1357.156	2	678.578	15.140	.000
	Order 7	2532.156	2	1266.078	24.990	.000
	Order 8	134.119	2	67.059	1.178	.314
	Order 9	814.827	2	407.413	8.265	.001
	Order 10	459.344	2	229.672	3.224	.046
	Order 11	93.692	2	46.846	.863	.426
	Order 12	294.442	2	147.221	2.481	.091
	Order 13	646.188	2	323.094	6.294	.003
	Order 14	120.504	2	60.252	1.092	.341
	Order 15	342.447	2	171.223	3.134	.050
	Order 16	132.932	2	66.466	1.052	.355
	Order 17	40.122	2	20.061	.298	.743
time * Temperature	Linear	458021.046	2	229010.523	3407.305	.000
	Quadratic	19883.323	2	9941.662	172.750	.000
	Cubic	141056.936	2	70528.468	1257.199	.000
	Order 4	10901.156	2	5450.578	90.674	.000
	Order 5	12436.344	2	6218.172	90.208	.000
	Order 6	6048.634	2	3024.317	67.476	.000
	Order 7	3220.967	2	1610.484	31.787	.000
	Order 8	1013.092	2	506.546	8.897	.000
	Order 9	920.591	2	460.296	9.338	.000
	Order 10	4039.710	2	2019.855	28.350	.000
	Order 11	147.770	2	73.885	1.361	.263
	Order 12	4558.373	2	2279.186	38.403	.000
	Order 13	6536.427	2	3268.213	63.668	.000
	Order 14	4975.919	2	2487.960	45.083	.000
	Order 15	5648.904	2	2824.452	51.701	.000
	Order 16	1879.274	2	939.637	14.868	.000
	Order 17	312.337	2	156.169	2.319	.106
time * Location * Humidity	Linear	8741.317	6	1456.886	21.676	.000
	Quadratic	19433.674	6	3238.946	56.281	.000
	Cubic	5211.589	6	868.598	15.483	.000
	Order 4	6898.182	6	1149.697	19.126	.000
	Order 5	5378.726	6	896.454	13.005	.000
	Order 6	2749.086	6	458.181	10.223	.000
	Order 7	4440.521	6	740.087	14.608	.000
	Order 8	1286.362	6	214.394	3.766	.003
	Order 9	3520.144	6	586.691	11.902	.000
	Order 10	1114.411	6	185.735	2.607	.024
	Order 11	5466.376	6	911.063	16.784	.000
	Order 12	1398.780	6	233.130	3.928	.002
	Order 13	1173.370	6	195.562	3.810	.002
	Order 14	1973.894	6	328.982	5.961	.000
	Order 15	506.811	6	84.468	1.546	.176

Tests of Within-Subjects Contrasts

Measure: MEASURE_1

Source	time	Type III Sum of Squares	df	Mean Square	F	Sig.
	Order 16	345.864	6	57.644	.912	.491
	Order 17	855.660	6	142.610	2.117	.062
time * Location * Temperature	Linear	12307.180	6	2051.197	30.518	.000
	Quadratic	73395.390	6	12232.565	212.558	.000
	Cubic	22416.824	6	3736.137	66.598	.000
	Order 4	22126.840	6	3687.807	61.349	.000
	Order 5	23001.775	6	3833.629	55.615	.000
	Order 6	5961.645	6	993.607	22.169	.000
	Order 7	4582.006	6	763.668	15.073	.000
	Order 8	5446.026	6	907.671	15.943	.000
	Order 9	1737.267	6	289.544	5.874	.000
	Order 10	1906.030	6	317.672	4.459	.001
	Order 11	902.567	6	150.428	2.771	.018
	Order 12	1159.603	6	193.267	3.256	.007
	Order 13	1572.672	6	262.112	5.106	.000
	Order 14	267.992	6	44.665	.809	.566
	Order 15	927.242	6	154.540	2.829	.016
	Order 16	1183.352	6	197.225	3.121	.009
	Order 17	1288.001	6	214.667	3.187	.008
time * Humidity * Temperature	Linear	14776.465	4	3694.116	54.962	.000
	Quadratic	18853.745	4	4713.436	81.902	.000
	Cubic	18996.586	4	4749.147	84.655	.000
	Order 4	6345.251	4	1586.313	26.389	.000
	Order 5	15259.518	4	3814.880	55.343	.000
	Order 6	7747.553	4	1936.888	43.214	.000
	Order 7	1633.367	4	408.342	8.060	.000
	Order 8	1889.932	4	472.483	8.299	.000
	Order 9	138.968	4	34.742	.705	.591
	Order 10	351.824	4	87.956	1.235	.304
	Order 11	298.677	4	74.669	1.376	.251
	Order 12	763.271	4	190.818	3.215	.017
	Order 13	894.943	4	223.736	4.359	.003
	Order 14	329.622	4	82.405	1.493	.213
	Order 15	898.073	4	224.518	4.110	.005
	Order 16	626.335	4	156.584	2.478	.052
	Order 17	97.350	4	24.338	.361	.835
time * Location * Humidity * Temperature	Linear	27681.533	12	2306.794	34.321	.000
	Quadratic	84948.759	12	7079.063	123.008	.000
	Cubic	28649.820	12	2387.485	42.558	.000
	Order 4	19694.051	12	1641.171	27.302	.000
	Order 5	11914.063	12	992.839	14.403	.000
	Order 6	9785.872	12	815.489	18.195	.000
	Order 7	5274.936	12	439.578	8.676	.000
	Order 8	3287.922	12	273.994	4.813	.000
	Order 9	3297.676	12	274.806	5.575	.000

Tests of Within-Subjects Contrasts

Measure: MEASURE_1

Source	time	Type III Sum of Squares	df	Mean Square	F	Sig.
	Order 10	1051.680	12	87.640	1.230	.280
	Order 11	3557.770	12	296.481	5.462	.000
	Order 12	2406.300	12	200.525	3.379	.001
	Order 13	1887.730	12	157.311	3.065	.002
	Order 14	4066.128	12	338.844	6.140	.000
	Order 15	1508.019	12	125.668	2.300	.015
	Order 16	1464.680	12	122.057	1.931	.044
	Order 17	3031.515	12	252.626	3.751	.000
Error(time)	Linear	4839.237	72	67.212		
	Quadratic	4143.558	72	57.549		
	Cubic	4039.178	72	56.100		
	Order 4	4328.038	72	60.112		
	Order 5	4963.068	72	68.931		
	Order 6	3227.083	72	44.821		
	Order 7	3647.825	72	50.664		
	Order 8	4099.074	72	56.932		
	Order 9	3549.060	72	49.292		
	Order 10	5129.803	72	71.247		
	Order 11	3908.207	72	54.281		
	Order 12	4273.136	72	59.349		
	Order 13	3695.938	72	51.332		
	Order 14	3973.405	72	55.186		
	Order 15	3933.393	72	54.630		
	Order 16	4550.164	72	63.197		
	Order 17	4849.254	72	67.351		

Tests of Between-Subjects Effects

Measure: MEASURE_1

Transformed Variable: Average

Source	Type III Sum of Squares	df	Mean Square	F	Sig.
Intercept	90033224.06	1	90033224.06	1230112.253	.000
Location	298742.911	3	99580.970	1360.562	.000
Humidity	54054.902	2	27027.451	369.273	.000
Temperature	642356.265	2	321178.132	4388.215	.000
Location * Humidity	139277.440	6	23212.907	317.155	.000
Location * Temperature	94025.932	6	15670.989	214.111	.000
Humidity * Temperature	6908.168	4	1727.042	23.596	.000
Location * Humidity * Temperature	159726.287	12	13310.524	181.860	.000
Error	5269.757	72	73.191		
