# Supplementary material for: Construction of a Novel Degradation Model of Bacillus thuringiensis Protein in Soil and Its Application in Estimation of the Degradation Dynamics of Bt-Cry1Ah Protein
Source: Front Plant Sci. 2022 Apr 13;13:875020. doi: 10.3389/fpls.2022.875020 (PMC9043894; doi:10.3389/fpls.2022.875020)
Supplement: Supplementary Table S5 — The ANOVA of the degradation data of Cry1Ah protein under soil sterilization and non-sterilization conditions. [file Data_Sheet_2.PDF]

```
GLM h0 h4 h8 h12 h16 h20 h24 d1.5 d2 d3 d4 d7 d11 d16 d21 d32 d64 d96 BY s
  /WSFACTOR=time 18 Polynomial
  /METHOD=SSTYPE(3)
  /CRITERIA=ALPHA(.05)
  /WSDESIGN=time
  /DESIGN=s.
```

General Linear Model

Notes

Output Created		15-OCT-2020 10:35:24
Comments		
Input	Data	C:\360CloudEnterprise\Cache\557795417\14829270102172675\周蕾\数据\方差分析\灭菌数据.sav
	Active Dataset	数据集1
	Filter	<none>
	Weight	<none>
	Split File	<none>
	N of Rows in Working Data File	6
Missing Value Handling	Definition of Missing	User-defined missing values are treated as missing.
	Cases Used	Statistics are based on all cases with valid data for all variables in the model.
Syntax		GLM h0 h4 h8 h12 h16 h20 h24 d1.5 d2 d3 d4 d7 d11 d16 d21 d32 d64 d96 BY s /WSFACTOR=time 18 Polynomial /METHOD=SSTYPE(3) /CRITERIA=ALPHA(.05) /WSDESIGN=time /DESIGN=s.
Resources	Processor Time	00:00:00.00
	Elapsed Time	00:00:00.06

[数据集1] C:\360CloudEnterprise\Cache\557795417\14829270102172675\周蕾\数据\方差分析\灭菌数据.sav

Within-Subjects Factors

Measure: MEASURE_1

time	Dependent Variable
1	h0
2	h4
3	h8
4	h12
5	h16
6	h20
7	h24
8	d1.5
9	d2
10	d3
11	d4
12	d7
13	d11
14	d16
15	d21
16	d32
17	d64
18	d96

Between-Subjects Factors

		N
s	non-ster	3
	steriliz	3

Multivariate Tests[a]

Effect		Value	F	Hypothesis df	Error df	Sig.
time	Pillai's Trace	.[b]	.	.	.	.
	Wilks' Lambda	.[b]	.	.	.	.
	Hotelling's Trace	.[b]	.	.	.	.
	Roy's Largest Root	.[b]	.	.	.	.
time * s	Pillai's Trace	.[b]	.	.	.	.
	Wilks' Lambda	.[b]	.	.	.	.
	Hotelling's Trace	.[b]	.	.	.	.
	Roy's Largest Root	.[b]	.	.	.	.

a. Design: Intercept + s
Within Subjects Design: time

b. Cannot produce multivariate test statistics because of insufficient residual degrees of freedom.

Mauchly's Test of Sphericity[a]

Measure: MEASURE_1

Within Subjects Effect	Mauchly's W	Approx. Chi-Square	df	Sig.	Epsilon[b]		
					Greenhouse-Geisser	Huynh-Feldt	Lower-bound
time	.000	.	152	.	.162	.692	.059

Tests the null hypothesis that the error covariance matrix of the orthonormalized transformed dependent variables is proportional to an identity matrix.

a. Design: Intercept + s
Within Subjects Design: time

b. May be used to adjust the degrees of freedom for the averaged tests of significance. Corrected tests are displayed in the Tests of Within-Subjects Effects table.

Tests of Within-Subjects Effects

Measure: MEASURE_1

Source		Type III Sum of Squares	df	Mean Square	F	Sig.
time	Sphericity Assumed	1497577.274	17	88092.781	2578.658	.000
	Greenhouse-Geisser	1497577.274	2.762	542222.359	2578.658	.000
	Huynh-Feldt	1497577.274	11.770	127242.049	2578.658	.000
	Lower-bound	1497577.274	1.000	1497577.274	2578.658	.000
time * s	Sphericity Assumed	5983.644	17	351.979	10.303	.000
	Greenhouse-Geisser	5983.644	2.762	2166.476	10.303	.002
	Huynh-Feldt	5983.644	11.770	508.402	10.303	.000
	Lower-bound	5983.644	1.000	5983.644	10.303	.033
Error(time)	Sphericity Assumed	2323.034	68	34.162		
	Greenhouse-Geisser	2323.034	11.048	210.273		
	Huynh-Feldt	2323.034	47.078	49.344		
	Lower-bound	2323.034	4.000	580.758		

Tests of Within-Subjects Contrasts

Measure: MEASURE_1

Source	time	Type III Sum of Squares	df	Mean Square	F	Sig.
time	Linear	1344617.662	1	1344617.662	41280.011	.000
	Quadratic	117782.372	1	117782.372	4623.666	.000
	Cubic	2403.148	1	2403.148	37.923	.004
	Order 4	25844.467	1	25844.467	625.304	.000
	Order 5	2858.474	1	2858.474	794.848	.000
	Order 6	5.429	1	5.429	.124	.742
	Order 7	594.806	1	594.806	56.850	.002
	Order 8	.074	1	.074	.018	.901
	Order 9	86.613	1	86.613	2.182	.214
	Order 10	1625.596	1	1625.596	1457.442	.000
	Order 11	90.968	1	90.968	2.384	.197
	Order 12	1303.101	1	1303.101	105.857	.001
	Order 13	30.649	1	30.649	2.491	.190
	Order 14	130.008	1	130.008	1.710	.261
	Order 15	2.303	1	2.303	.029	.874
	Order 16	119.274	1	119.274	2.910	.163
	Order 17	82.331	1	82.331	1.483	.290
time * s	Linear	4393.487	1	4393.487	134.881	.000
	Quadratic	463.296	1	463.296	18.187	.013
	Cubic	167.868	1	167.868	2.649	.179
	Order 4	55.950	1	55.950	1.354	.309
	Order 5	357.492	1	357.492	99.407	.001
	Order 6	59.655	1	59.655	1.368	.307
	Order 7	14.723	1	14.723	1.407	.301
	Order 8	32.027	1	32.027	7.686	.050
	Order 9	8.567	1	8.567	.216	.666
	Order 10	.218	1	.218	.195	.682
	Order 11	5.973	1	5.973	.157	.713
	Order 12	30.826	1	30.826	2.504	.189
	Order 13	13.942	1	13.942	1.133	.347
	Order 14	185.079	1	185.079	2.434	.194
	Order 15	26.829	1	26.829	.335	.594
	Order 16	164.965	1	164.965	4.025	.115
	Order 17	2.748	1	2.748	.049	.835
Error(time)	Linear	130.292	4	32.573		
	Quadratic	101.895	4	25.474		
	Cubic	253.480	4	63.370		
	Order 4	165.324	4	41.331		
	Order 5	14.385	4	3.596		
	Order 6	174.480	4	43.620		
	Order 7	41.851	4	10.463		

Tests of Within-Subjects Contrasts

Measure: MEASURE_1

Source	time	Type III Sum of Squares	df	Mean Square	F	Sig.
	Order 8	16.667	4	4.167		
	Order 9	158.762	4	39.690		
	Order 10	4.462	4	1.115		
	Order 11	152.637	4	38.159		
	Order 12	49.240	4	12.310		
	Order 13	49.216	4	12.304		
	Order 14	304.144	4	76.036		
	Order 15	320.175	4	80.044		
	Order 16	163.927	4	40.982		
	Order 17	222.097	4	55.524		

Tests of Between-Subjects Effects

Measure: MEASURE_1

Transformed Variable: Average

Source	Type III Sum of Squares	df	Mean Square	F	Sig.
Intercept	5904408.612	1	5904408.612	110967.211	.000
s	5858.710	1	5858.710	110.108	.000
Error	212.834	4	53.209		
